# Latest advances in the management of classical Hodgkin lymphoma: the era of novel therapies

**DOI:** 10.1038/s41408-021-00518-z

**Published:** 2021-07-09

**Authors:** Razan Mohty, Rémy Dulery, Abdul Hamid Bazarbachi, Malvi Savani, Rama Al Hamed, Ali Bazarbachi, Mohamad Mohty

**Affiliations:** 1 Division of Hematology and Oncology, Department of Internal Medicine, American University of Beirut Medical Center, Beirut, Lebanon; 2 Department of Hematology, Saint Antoine Hospital, AP-HP, Paris, France; 3 Sorbonne University, INSERM UMRs 938, Centre de Recherche Saint-Antoine (CRSA), Paris, France; 4 Department of Internal Medicine, Jacobi Medical Center, Albert Einstein College of Medicine, New York, NY USA; 5 Division of Hematology and Oncology, Department of Medicine, University of Arizona Cancer Center, Tucson, AZ USA

**Keywords:** Targeted therapies, Immunosurveillance

## Abstract

Hodgkin lymphoma is a highly curable disease. Although most patients achieve complete response following frontline therapy, key unmet clinical needs remain including relapsed/refractory disease, treatment-related morbidity, impaired quality of life and poor outcome in patients older than 60 years. The incorporation of novel therapies, including check point inhibitors and antibody–drug conjugates, into the frontline setting, sequential approaches, and further individualized treatment intensity may address these needs. We summarize the current treatment options for patients with classical Hodgkin lymphoma from frontline therapy to allogeneic hematopoietic stem cell transplantation and describe novel trials in the field.

## Introduction

Decade after decade, the prognosis of classical Hodgkin lymphoma (cHL) has improved with the advancement of novel treatment strategies [1] resulting in high cure rates. In early stage disease, using the European Organization for Research and Treatment of Cancer staging criteria, estimates of 5-year overall survival (OS) range from 99.4 to 96.0%, for favourable and unfavourable risk, respectively, a small but significant difference (*p* < 0.001) [2]. In the advanced-stage disease, 5-year OS ranges from 56 to 89% [3]. However, key unmet clinical needs remain. New treatment strategies are necessary to prevent or cure relapsed/refractory (R/R) disease, reduce treatment-related morbidity, ameliorate quality of life (QoL), and improve outcomes of patients older than 60 years. This review aims to summarize the most important advances in cHL management to address these needs in both frontline and relapse settings.

## Frontline therapy in Hodgkin lymphoma

### Risk-adapted and response-adapted frontline strategies

Most patients with cHL will be cured with standard treatment. However, they are at risk of potential long-term complications including the exponential increase in cardiopulmonary toxicities and secondary malignancies as well as QoL impairment [4]. Consequently, the latest advances in the management of cHL have focused on optimizing treatment strategies to improve outcome while reducing toxicity. Identifying patients at low or high risk of recurrence is critical to avoid over- or under-treatment. The fluorodeoxyglucose positive emission tomography (PET) scan has become an essential tool for staging and therapeutic guidance [5–7]. The predictive power of PET can also be improved by assessing the metabolic tumour volume (MTV) measured by drawing the volume of fluorodeoxyglucose-avid disease in three dimensions [8, 9].

The standard of care for early stage cHL is doxorubicin (or adriamycin), bleomycin, vinblastine, and dacarbazine (ABVD) and form the backbone of frontline management in North America regardless of the stage. The benefit of adding radiation therapy was tested in the early favourable setting by the HD10 trial showing that the addition of 20 Gy of involved-field radiotherapy (IFRT) to 2 cycles of ABVD is as effective as 30 Gy [10]. In the RAPID trial, patients who had a negative PET after 3 cycles of ABVD had a very good prognosis whether consolidation radiation therapy was given or not with an absolute difference in the 3-year progression free survival (PFS) of -3.8% (95% confidence interval (CI), −8.8 to 1.3) [11]. The H10 EORTC/Lysa trial demonstrated the benefit of a PET-adapted strategy after 2 cycles of ABVD in favourable and unfavourable early stage cHL. Patients either received 1 additional cycle of ABVD with involved-node radiation therapy (INRT) regardless of PET results (standard arm) or stratified to receive additional ABVD in PET negative patients or escalated (e)BEACOPP (bleomycin, etoposide, doxorubicin, cyclophosphamide, vincristine (or oncovin), procarbazine, prednisolone) with INRT in PET positive patients. Switching to eBEACOPP with INRT significantly improved 5-year PFS from 77.4% in the standard arm to 90.6% in the intensification arm (Hazard ratio [HR] = 0.42; 95%CI, 0.23–0.74; *P* = .002). The omission of INRT lead to inferior outcome in PET negative patients compared to the standard arm especially in favourable risk patients who received a total of four ABVD cycles (HR, 1.45; 95% CI, 0.8–2.5). Long-term follow-up from the German Hodgkin Study Group (GHSG) HD14 randomized trial including young patients showed improved disease control with two cycles of eBEACOPP, followed by two cycles of ABVD (2 + 2) compared with four cycles of ABVD alone (4xABVD). Patients receiving 2 + 2 had higher 10-year PFS of 91.2% compared to 85.6% with 4xABVD (HR = 0.052, 95%CI, 0.386–0.704, *p* < 0.0001). However, this did not translate into an OS difference. Therefore, 2 + 2 is the GHSG standard of care for patients aged ≤60 years with early unfavourable cHL. Yet, this approach is not widely used. Higher toxicities associated with the eBEACOPP regimen including short- and long-term toxicities namely haematologic malignancies makes it less convenient. (Table 1) [12, 13].

**Table 1:** Summary of phase I, II and III trials and retrospective studies in Hodgkin Lymphoma.

Trial	Design	Study population	Study arms	Outcome
*Frontline*				
NU16H08 [74]	Phase II	> 18 years *N* = 30	PEM followed by AVD	After PEM: 37%, CMR 25%, >90% decrease MTV on PET-CT After AVD: All, CMR
NIVAHL [23]	Phase II	18–60 years *N* = 110	Arm A: Nivo+AVD x4 Arm B: Nivo x4 then Nivo+AVD x2 then AVD x2	ORR: Arm A: 100%, B: 96% CR: Arm A: 81%, B: 86% 1-year PFS: 98% 1-year OS: 100%
Trial of the LYSA group [28]	Phase II	> 60 years *N* = 89	PVAB x6	CMR: 77% 2-year OS: 84% 2-year PFS: 61%
HD14 [12]	Randomized parallel arm trial	< 60 years Early stage, unfavourable N = 1112	BEACOPP x2 then ABVD x2 Or ABVD x4	10-years PFS: 91.2 vs 85.6%, *p* < 0.0001 10-years OS: 94% vs 94.1
NCT01716806 [41]	Phase II	> 60 years *N* = 19	BV + Nivo < 16 cycles	ORR: 100%, CR rate: 72%, PR rate: 28%
*Salvage therapy*				
Stamatoullas et al. [35]	Phase I/II	*N* = 42	BV-ICE x2 then if CMR BV-ICE x1 then BV x1 then ASCT	CMR: 69.2%, PR: 25.6% 1-year PFS: 69%
Herrera et al. [36]	Phase II	*N* = 39	Nivo x6 If CR - > ASCT If no CR - > Nivo-ICE x2	1-year OS: 97% 1-year PFS: 79%
Moskowitz et al. [37]	Phase II	*N* = 39	PEM-GVHD x2-4 then ASCT	ORR: 100%, CR rate: 95%, PR rate: 5% No relapse or death (median follow-up 11.2 months)
Herrera et al. [39, 40]	Phase I/II	≥18 years *N* = 61	BV + Nivo x4 +/- ASCT	ORR: 85%, CR: 67% 2-year PFS: 78% (if ASCT, 91%) 2-year OS: 93%
LaCasce [38]	Phase I/II	*N* = 53	BvB <6 cycles then ASCT or BV x14	CR: 95% (if ASCT, 94%) 2-year PFS: 69.8% (if ASCT, 63%)
Checkmate 205 [75]	Phase II	*N* = 276	Nivo after ASCT Cohort A: BV naïve *N* = 60 Cohort B: Failure of BV post-ASCT *N* = 60 Cohort C: After BV before and/or after ASCT failure	ORR/CR: Overall: 69%/16% Cohort A: 65%/29% Cohort B: 68%/13% Cohort C: 73%/12% Median PFS: Overall: 14.7 m Cohort A: 18.3 m Cohort B: 14.7 m Cohort C: 11.9 m
*Primary refractory disease*				
KEYNOTE-024 [58]	Phase III	≥18 years *N* = 304	PEM vs BV	mPFS: 13.2 vs 8.3 m
*CPI before allo-HCT*				
Merryman et al. [62, 76]	Retrospective	Pts treated with anti-PD1 mAb before transplant *N* = 150	TT: Nivo *N* = 118, PEM *N* = 31, avelumab *N* = 1 Transplant: Haplo (47%), MSD (19%), MUD (26%), MMUD (5%), CB (1%), unknown (1%)	2-year PFS: 65% 2-year OS: 79% 2-year NRM: 14% 6-m CI of grade 2-4 aGVHD: 39% 6-m CI of grade 3-4 aGVHD: 16% 2-year CI of cGVHD: 45%
Manson et al. [62]	Retrospective	Pts treated with Nivo with a cohort of allo-SCT *N* = 17	TT: Nivo Transplant	1-year PFS: 76% 1-year OS: 82% 1-year TRM: 17.6% Grade 2-4 aGVHD: 82% cGVHD: 29%
El Cheikh et al. [77]	Retrospective	Nivo followed by allo-SCT *N* = 9	TT: Nivo *N* = 9 Transplant: Haplo (67%) MSD (33%)	Grade 2-4 aGVHD: 100% cGVHD: 33%
*CPI for relapse after allo-HCT*				
Herbaux et al. [69]	Retrospective	Relapse after allo-SCT *N* = 20	Nivo	ORR: 95%, CR: 42%, PR: 52% 1-year PFS: 58% 1-year OS:79% GvHD: 30% of pts
Haverkos et al. [70]	Retrospective	Relapse after allo-SCT *N* = 31	Nivo or PEM	GvHD: 55% of pts (59% aGvHD, 41% cGvHD)
*Ibrutinib for relapse after allo-SCT*				
Badar et al. [71]	Retrospective	≥18 years *N* = 7	Ibrutinib	ORR: 57%, CR: 43%, PR: 14%

*PEM* pembrolizumab, *AVD* Adriamycin, Vincristine, Dacarbazine, *CMR* complete metabolic response, *MTV* metabolic tumour volume, *PET-CT* positron emission tomography/computed tomography, *Nivo* Nivolumab, *PFS* progression free survival, *OS* overall survival, *PVAB* prednisone, vinblastine, doxorubicin and bendamustine, *ABVD* Adriamycin, Bleomycin, Vincristine, Dacarbazine, *BV* brentuximab vedotin, *ORR* overall response rate, *CR* complete response, *PR* partial response, *ICE* ifosfamide, mesna, carboplatin, etoposide, *ASCT* autologous stem cell transplantation, *BvB* brentuximab vedotin, bendamustin, *mPFS* median progression free survival, *m* months, *CPI* check point inhibitors, *allo-HCT* allogeneic hematopoietic stem cell transplantation, *TT* treatment, *Haplo* haploidentical transplant, *MSD* matched sibling donor, *MUD* matched unrelated donor, *MMUD* mismatched unrelated donor, *CI* cumulative incidence, *GVHD* graft versus host disease, *aGVHD* acute GVHD, *cGVHD* chronic GVHD, *pts* patients, *eBEACOPP* bleomycin, etoposide, doxorubicin (aka adriamycin), cyclophosphamide, vincristine (aka oncovin), procarbazine, prednisolone.

In the HD17 phase 3 trial, 1100 patients were randomized to receive either 2 + 2 followed by 30 Gy IFRT or PET4-guided treatment using 2 + 2 regimen followed by 30 Gy IFRT only in patients with positive end of treatment PET. Five-year PFS was 97.3% in the standard arm and 95.1% in the PET4-guided treatment group (HR = 0.523, 95%CI 0.226–1.211). This study shows for the first time that consolidation radiotherapy can safely be omitted without significant loss of efficacy in newly diagnosed early stage unfavourable cHL in PET4 negative patients receiving 2 + 2 chemotherapy, thus reducing the proportion of patients at risk of late toxic effects of radiotherapy [14].

In advanced-stage cHL, the HD18 trial demonstrated the non-inferiority of reducing therapy to a total of four cycles of eBEACOPP instead of six or eight cycles in the case of PET2 negativity after two cycles of eBEACOPP (5-year PFS 92.2% vs 90.8%, respectively, 95% CI−2·7–5·4) [15]. Also, the AHL2011 Lysa trial validated an alternative PET-adapted approach after two cycles of eBEACOPP in patients with advanced cHL. Patients with PET2-negative disease received four additional cycles of ABVD and patients with PET2-positive disease received four additional cycles of eBEACOPP leading to similar 4-year PFS 87.1% vs 87.4%, respectively (*p* = 0.68) and decrease toxicity in the PET2-negative arm, mainly cytopenia and sepsis [16, 17]. Another PET-directed therapeutic strategy was studied in the RATHL trial, which included 1214 patients with newly diagnosed advanced cHL [18]. After two cycles of ABVD, PET negative patients were randomized to receive either four cycles of AVD, omitting bleomycin, or ABVD. Progressing patients received eBEACOPP. Three-years PFS was comparable in the AVD (84.4%) and ABVD (85.7%) groups. Three-years OS was also similar, 97.6% and 97.2%, respectively. These results offer a PET-adapted approach reducing bleomycin exposure, which translates into a lower incidence of pulmonary toxicity without compromising efficacy [18].

### Immunotherapy as frontline therapy

The risk-adapted and response-adapted approaches discussed above rely mainly on intensification or de-escalation chemotherapy. More recent approaches combine novel immunotherapies such as brentuximab vedotin (BV) and anti-PD-1 monoclonal antibodies (mAb) to reduce the risk of relapse and chemotherapy-associated toxicity. BV, an antibody–drug conjugate that selectively targets tumour cells expressing the CD30 antigen, was initially added to ABVD and subsequently replaced bleomycin to avoid pulmonary toxicity [19]. The ECHELON-1 randomized phase III trial compared six cycles of ABVD to six cycles of BV plus AVD as frontline treatment for 1334 patients with advanced cHL. The 3-year modified PFS was moderately improved with BV-AVD (83.1% versus 76% with ABVD) [20]. This benefit of BV-AVD was confirmed at 5 years with a PFS of 82.2% compared to 75.3% with ABVD (HR = 0.681, *p* = 0.002) [21]. However, no significant difference was observed in terms of OS. Peripheral neuropathy, neutropenia and infections were more frequent in the BV-AVD arm reduced with the use of prophylactic granulocyte colony-stimulating factor while pulmonary complications were lower in the BV-AVD. Hence, BV-AVD is a safe new frontline option for patients with advanced-stage cHL allowing durable efficacy without the need for treatment intensification or bleomycin exposure.

The use of sequential pembrolizumab (PEM) and AVD for untreated early unfavourable or advanced-stage cHL was evaluated in a phase II study. Thirty patients were treated sequentially with three cycles of PEM followed by four to six cycles of AVD chemotherapy based on the initial stage with no consolidative radiotherapy. Following PEM monotherapy, 11 (37%) patients achieved a complete metabolic response (CMR), and 7 of 28 (25%) patients had >90% reduction of the MTV. Following two cycles of AVD, 100% of the patients had a sustained CMR. After a median follow-up of 22.5 months, there have been no changes in therapy, progression, or death and treatment was well tolerated. PFS and OS rates were 100%. Overall, sequential PEM and AVD were safe and highly active in this population [22].

Nivolumab (nivo) is highly effective in R/R cHL but has not been adequately studied in frontline therapy. A multicentre, phase II, randomized NIVAHL trial evaluated the efficacy of two experimental Nivo-based first-line treatment strategies [23]. Early stage unfavourable cHL patients aged 18–60 years were included. Randomization was to either Arm A: concomitant systemic treatment with four cycles of Nivo and AVD (Nivo-AVD), or Arm B: sequential treatment with four doses of Nivo followed by two cycles of Nivo-AVD and two cycles of AVD at standard doses. Both groups then received 30-Gy involved-site radiotherapy (ISRT). Serious adverse events (AE) occurred in 38% and 28% of Arm A and Arm B, respectively. Treatment-related morbidity defined as grade ≥3 organ toxicity, or anaemia, thrombocytopenia, or infection of grade 4 was documented in 16% and 22% of patients, respectively. At the first interim restaging after two cycles of Nivo-AVD or four cycles of Nivo, the overall response rate (ORR) was 100% and 96%, and the complete response (CR) rate was 85% and 53%, respectively. At the end of systemic treatment, the ORR was 100% and 96%, and the CR rate was 81% and 86%, respectively. The 1-year PFS and OS were 98% and 100% and the 2-year PFS and OS were 98% and 100%, respectively [23, 24]. Overall, concomitant and sequential therapy with Nivo-AVD is feasible with acceptable toxicity, high early CR rate and promising 2-year PFS.

In summary, the addition of immunotherapies to established chemotherapy results in improved outcomes. In terms of toxicity, these new advances make it possible to avoid the toxicity of bleomycin and even radiotherapy in some cases. However, these new agents can induce immune-related AEs or peripheral neuropathy. To assess the benefit and optimal use of immunotherapies as a frontline treatment for patients with cHL, further investigations in randomized trials are warranted. The results of the ongoing phase III trial (NCT03907488) comparing Nivo-AVD to BV-AVD are of particular interest.

### Novel reduced-toxicity approaches for elderly patients

Patients aged >60 years represent 20–30% of all cHL. The disease is usually aggressive and characterized by unfavourable prognostic factors and poor tolerance to chemotherapy resulting in significantly reduced survival compared to younger patients. In R/R cHL, prospective and retrospective studies have shown that bendamustine monotherapy provides interesting efficacy and an acceptable toxicity profile [25–27]. A combination regimen of prednisone, vinblastine, doxorubicin and bendamustine (PVAB) for first-line therapy of older patients showing high CMR, 2-year OS, 2-year PFS were 77%, 84% and 61%, respectively, with an acceptable toxicity profile, a particularly favourable outcome but long-term follow-up for survival is needed (Table 1) [28].

As an attempt to decrease chemotherapy exposure in elderly cHL patients, BV and anti-PD-1 mAb are now combined with chemotherapy or administered as a single agent. A promising approach is the sequential administration of two cycles of BV alone followed by six cycles of AVD and four consolidative doses of BV in responding patients. Forty-eight patients with a median age of 68 years were included in a phase II trial. Thirty-seven (77%) patients completed six cycles of AVD and 35 (73%) received at least one BV consolidative dose. The ORR and CR rates were 82% and 36% after initial BV, and 95% and 90% after AVD, respectively. This approach was well tolerated and allowed 2-year PFS and OS rates of 84% and 93%, respectively [29].

A chemotherapy-free combination of BV-Nivo was also evaluated in two phase II trials as frontline therapy in elderly patients. In the ACCRU study, 46 patients received 8 cycles of BV-Nivo. The trial was closed after the interim analysis failed to meet the predefined criteria, however, the regimen was well tolerated and showed an ORR of 61% with 48% CMR. After a median follow-up of 21.2 months, the median PFS was 18.3 months, and the median OS was not reached. Another trial assessed the BV-Nivo combination in 21 patients. After a median follow-up of 26.2 months, the ORR was 95% and the median PFS was not reached [30]. For more fragile, unfit, elderly patients, BV alone or in combination with dacarbazine may also represent well-tolerated and beneficial approaches [30, 31].

Overall, the incorporation of immunotherapy in the frontline treatment for older cHL patients is a feasible and promising strategy. Further comparative studies are needed, however, to evaluate the benefit of these novel agents in terms of QoL and OS in this particular population.

## Salvage therapy for relapsed Hodgkin lymphoma

About 10–15% of patients with early stage and 15-30% with advanced-stage cHL fail to respond or relapse after primary conventional treatment. Despite the approval of novel therapies, autologous stem cell transplantation (ASCT) remains the standard of care in these patients. However, data supporting the benefits of ASCT dates back to two small studies published in 1993 and 2002 [32, 33]. As the disease status before ASCT is the most important factor predicting outcome, second-line therapy must induce a high response. BV has shown significant activity in phase II single arm, multicentre pivotal study in patients with R/R cHL [34]. Based on these results, BV was used in combination with chemotherapy in a phase I/II trial of the LYSARC (Lymphoma Academic Research Organisation). Patients received two cycles of BV-ICE (ifosfamide, carboplatin, etoposide) followed by PET evaluation. Only those with CMR received a third course of treatment followed by 1 cycle of single agent BV prior to ASCT. Patients who did not achieve CMR received off-study treatment. Most of the patients (*n* = 27, 69.2%) had a CMR and 20 underwent ASCT. During follow up, 13 patients relapsed, and no death was observed without progression. The 1-year PFS and OS were 69% and 100%, respectively [35].

An innovative sequential approach using Nivo was evaluated in a phase II trial. Nivo was administered for a maximum of six cycles as first-salvage therapy. Patients who achieved CR proceeded to ASCT while those who did not achieve CR received Nivo-ICE for two cycles. The 1-year PFS and OS were 79% and 97%, respectively. In this cohort, Nivo alone was an effective bridge to ASCT in most patients, sparing the toxicity of traditional chemotherapy. Patients who did not achieve CR with Nivo were effectively salvaged by Nivo-ICE, a well-tolerated and effective first-salvage approach [36].

Another encouraging strategy is the combination of PEM with gemcitabine, vinorelbine and liposomal doxorubicin including 39 patients with relapsed cHL. Patients who achieved CR by PET after two or four cycles proceeded to ASCT. Among 37 evaluable patients, 35 (95%) achieved CR after two (*n* = 34) or four cycles (*n* = 1) and 35 patients underwent ASCT. After a median follow-up of 11.2 months, no relapse or death occurred [37].

The combination of BV and bendamustine (BV-Benda) may also be highly active with manageable toxicity as first-salvage therapy especially for fragile patients. A phase I study included 53 patients who received up to six cycles of BV-Benda. Patients could proceed to ASCT at any time after cycle 2 and most patients did so after just two cycles of therapy. After a median of two cycles, the ORR was 92.5% with 39 (74%) patients achieving CR. Forty patients underwent ASCT. Thirty-one patients (25 of whom received ASCT) received consolidation with BV monotherapy. After a median follow-up of 21 months, the estimated 2-year PFS was 69.8% and 63% for patients who received ASCT and for all patients, respectively. Duration of CR was similar among patients who did (95%) and did not (94%) undergo ASCT [38].

### What if salvage therapy could be chemotherapy-free?

In a phase I/II study, patients with R/R cHL received up to four cycles of BV-Nivo as first-salvage therapy followed by ASCT. The ORR among 61 treated patients was 85%, with a CR rate of 67%. Higher responses were seen with higher CD30 + expression. Prior to ASCT, AEs occurred in 98% of patients, mostly grades 1 and 2. A total of 67 (74%) patients underwent ASCT. At a median of 22.6 months, 2-year PFS and OS were 78% and 93% for all patients, respectively. The 2-year PFS was 91% for those who underwent ASCT. The BV-Nivo combination showed tolerability, high CR rates and durable remissions among patients with R/R cHL, potentially providing those patients with an attractive alternative chemotherapy-free regimen [39, 40]. This combination was also shown to be both feasible and efficient in patients >60 years [41].

### Maintenance strategies after ASCT in high-risk patients

Up to 50% of patients relapse after ASCT, hence, maintenance strategies were developed to prevent or delay progression, more particularly in high-risk patients. The AETHERA trial demonstrated for the first time the benefit of BV maintenance after ASCT for up to 16 cycles. The study included 329 patients with primary R/R cHL within 1 year of initial therapy or extranodal relapse. The 5-year PFS was 59% with BV versus 41% with placebo (HR 0.521, 95% CI 0.379–0.717) [42]. Although peripheral neuropathy occurred in 67% of the patients [43], this AE improved and/or resolved in 90% of patients and did not meaningfully impact QoL [44]. No benefit in terms of OS however, has been reported, most likely due to the crossover effect of patients relapsing in the placebo arm and receiving BV subsequently. A shorter duration of BV maintenance (four cycles) after ASCT was tested by Kort et al. with 2-year PFS and OS of 72% and 100%, respectively [45].

Anti-PD1 mAb might also become a new standard for maintenance after ASCT, allowing a schedule twice as short than in the AETHERA trial. A phase II study evaluated PEM administered after ASCT for up to eight cycles in 30 patients with R/R cHL. Toxicities were manageable and the 18-month PFS was 82% [46]. More recently, data of eight cycles of BV-Nivo combination for post-ASCT maintenance were presented at the 2020 ASH meeting. The 18-month PFS was 92% with an acceptable safety profile [47]. Nevertheless, randomized comparative trials are warranted to confirm the benefit of post-ASCT maintenance using anti-PD1 mAb.

## Allogeneic hematopoietic cell transplantation

Although most of the cHL patients can be cured with first- and second-line therapy, some patients may still relapse or progress after intensive chemotherapy and ASCT, and thus, carry a poor prognosis. Most salvage therapeutic options now incorporate the use of BV or anti-PD1 mAb, either as monotherapy or in combination. Other promising approaches use the triplet combination of BV-Nivo with ipilimumab (a check point inhibitor [CPI]) [48], new antibody–drug conjugates such as camidanlumab tesirine [49, 50], AFM13 (an anti-CD30/CD16A-bispecific antibody) [51], or chimeric antigen receptor (CAR) T-cell therapy [52]. However, these new advances in the management of cHL have only been evaluated in small series with phase I/II, single arm studies and warrant further investigation.

Fit patients who relapse after ASCT may benefit from allogeneic hematopoietic cell transplantation (allo-HCT) with non-myeloablative conditioning (NMAC) or reduced-intensity conditioning (RIC). A retrospective multicentre study of 98 consecutive patients with cHL who underwent RIC or NMAC allo-HCT at 24 French and Belgian centers compared outcome with haploidentical (Haplo, *n* = 34), mismatched unrelated (MMUD, *n* = 27) or cord blood (CB, *n* = 37) donors. All patients in the Haplo group received T-cell replete NMAC transplant with post-transplant cyclophosphamide (PT-Cy). After a median follow-up of 31 months, the OS and PFS in the Haplo group were 75% and 66%, respectively, with no difference with the two other donor groups. The CI of grade 3–4 acute graft-versus-host disease (aGvHD) and chronic (c) GvHD were 3% and 15%, respectively, both being significantly lower in the Haplo cohort compared to the other groups. The non-relapse mortality (NRM) for the whole cohort was 12%. A significantly higher probability of GvHD-free, relapse-free survival (GRFS) was observed in patients who received Haplo (52% versus 22% and 31% in the CB and MMUD groups at 3 years, respectively, *p* = 0.02). The authors concluded that in the absence of an HLA-identical donor, a T-cell replete, NMAC Haplo with PT-Cy was associated with better outcomes compared to other alternative donors for patients with R/R cHL [53].

The question of the best donor type between Haplo (in the era of PT-Cy) and HLA-matched related donor (MRD) for patients with cHL undergoing allo-HCT is still being debated. A multicentre retrospective study analyzed the outcome of 151 cHL patients undergoing NMAC/RIC allo-HCT from Haplo (*n* = 61) or MRD (*n* = 90). In the Haplo group, OS, relapse incidence, and NRM were 81%, 21% and 9%, respectively, with no significant difference compared to the MRD group. Donor type had a significant impact on GRFS at 2 years (58% versus 42% in the Haplo and MRD groups, respectively, *p* = 0.03). In the multivariate analysis, MRD donors were independently associated with lower GRFS compared to Haplo donors (HR = 2.95, *p* < 0.001). Not achieving CR at time of allo-HCT was also associated with lower GRFS (HR = 1.74, *p* = 0.01). In summary, the Haplo PT-Cy platform significantly improved GRFS in patients receiving allo-HCT compared to MRD [54]. The favourable results with Haplo and PT-Cy were also confirmed in a large European Society for Blood and Marrow Transplantation (EBMT) study comprising 240 cHL patients. The 2-year OS and PFS were 72% and 57%, respectively [55].

## Immunotherapies as a bridge to allogeneic hematopoietic cell transplantation?

In a large EBMT study, BV was assessed as a bridge to allo-HCT in patients with R/R cHL compared to a group of no BV therapy prior to allo-HCT [56]. In multivariate analysis, pre-transplant BV had no significant effect on non-relapse mortality (NRM), relapse incidence, PFS, or OS, but significantly reduced the incidence of cGvHD. A French retrospective study looked at the use of BV monotherapy in patients with R/R cHL. Among the 145 patients that responded to BV, 54 patients received consolidation allo-HCT with a median PFS of 18.8 versus 8.7 months for the 91 patients without transplant (*p* < 0.0001). Those results from real-life settings confirm the importance of considering BV as a bridge to transplant and support the previously reported BV efficacy and manageable toxicity. Because of the short-term responses in most patients, the authors recommended that allo-HCT consolidation for responders should be considered as quickly as possible [57]. Results of a study comparing BV to PEM showed a significant improvement in median PFS among 304 R/R cHL patients receiving PEM versus BV (13.2 versus 8.3 months; HR = 0.65; 95%CI, 0.48–0.88, *p* = 0.0027). One-year PFS rates were 53.9% and 35.6%, respectively. The authors stated that PEM monotherapy should be standard of care for this patient population [58] and believe that BV will be used early in the disease course, not as monotherapy but in combination. However, several reports suggested that anti-PD-1 mAb might be associated with increased toxicity, notably severe aGvHD [59–61]. These results prompted a warning and raised challenging questions about the role, timing, optimal modalities, long-term efficacy and the need for consolidation allo-HCT after anti-PD-1 mAb. A retrospective study analyzed 78 patients with R/R cHL treated with Nivo in the French Early Access Program and compared their outcomes after subsequent allo-HCT. Among responding patients, none of the patients undergoing allo-HCT relapsed, whereas 62.2% of patients who were not consolidated with allo-HCT relapsed. Most patients treated with anti-PD-1 mAb monotherapy eventually progressed, notably those who did not achieve CR. Patients undergoing consolidation with allo-HCT after anti-PD-1 therapy experienced prolonged PFS compared with non-transplanted patients, but this difference did not translate into an OS benefit. This information should be considered when evaluating the risk/benefit ratio of allo-HCT after anti-PD-1 mAb [62].

Pre-transplant exposure to anti-PD-1 mAb may indeed improve PFS in patients who receive Haplo with PT-Cy. In a study of 59 cHL patients, outcomes based on pre-transplant exposure to anti-PD-1 mAb were compared. The 2-year OS and PFS were 77% versus 71% (*p* = 0.599) and 78% versus 53% (*p* = 0.066), respectively. The 2-year relapse/progression rate was 22% in the no-CPI cohort and 4% in the CPI cohort (*p* = 0.098). PD-1 blockade as a bridge to Haplo with PT-Cy did not increase toxicities or NRM, and CPI before Haplo seemed to improve PFS [63].

An important retrospective cohort study of 209 cHL patients undergoing allo-HCT after anti-PD-1 mAb was recently published. With a median follow-up of 24 months, the 2-year cumulative incidences of NRM and relapse were 14% and 18%, respectively; the 2-year GRFS, PFS and OS were 47%, 69% and 82%, respectively. The 180-day cumulative incidence of grade 3-4 aGvHD was 15% and the 2-year cumulative incidence of cGvHD was 34%. In the multivariable analysis, longer interval from anti-PD-1 mAbs to allo-HCT ( > 80 days) was associated with less frequent grade 3–4 aGvHD (HR = 0.4, p = 0.01), while additional treatment between anti-PD-1 mAb and allo-HCT was associated with a higher risk of relapse (HR = 2.9, p = 0.003). Notably, PT-Cy was associated with significant improvements in PFS and GRFS. The authors conclude that allo-HCT after anti-PD-1 mAb is associated with favourable outcomes. Their data strongly suggest that anti-PD-1 mAb impacts the post-allo-HCT course both in terms of toxicity and efficacy. Most notably, PT-Cy may represent the optimal transplant strategy for this patient population [64].

### Why does PT-Cy prophylaxis improve outcomes?

A recent study of 18 patients showed that the anti-PD-1 mAb Nivo can persist in plasma after transplantation for up to 56 days. Nivo binds and blocks anti-PD-1 on allogeneic T-cells and generates an increased T-cell activation. Nivo exposed patients had a higher incidence of severe GvHD, and a higher expression of effector T-cells compared with anti-PD-1-naive patients. However, patients receiving PT-Cy showed a similar risk of aGvHD and T-cell profile irrespective of the previous Nivo exposure. This T-cell activation status can be mitigated with the use of PT-Cy, thus reducing the risk of aGvHD [65].

With respect to the use of anti-PD-1 mAbs before allo-HCT, conclusions are as follows:

- For patients with R/R cHL, the use of anti-PD-1 mAbs until progression seems to be suboptimal when a curative option can be considered: a consolidative strategy with allo-HCT should be discussed for all responding patients.
- Allo-HCT performed after anti-PD-1 therapy is a feasible strategy associated with an excellent PFS and a low cumulative incidence of relapse.
- Patients undergoing allo-HCT after anti-PD-1 therapy experience prolonged PFS compared with non-transplanted patients.
- PT-Cy is associated with improved outcomes and reduced risk of GvHD: this GvHD prophylaxis should be considered for all cHL patients undergoing allo-HCT after anti-PD1 mAbs, even in the case of MMUD and MRD.

## Immunotherapies for Relapse after Allogeneic HCT

Although long-term disease control after allo-HCT can be achieved, post-transplant relapse is still common. One of the earliest immunotherapeutic modalities used for relapse after allo-HCT is donor lymphocyte infusion (DLI). Peggs et al. reported the outcome after DLI infusion for relapsed cHL after allo-SCT. The ORR was 56% in all patients. Four patients died of severe GHVD [66]. In a retrospective study, DLI was combined to bendamustin for the treatment of nine patients with relapsed cHL after allo-HCT. The ORR was 55% with 30% of them having CR. Three patients developed grade IV aGVHD and three patients developed cGVHD [67]. In a report by the lymphoma working party of the EBMT, Bazarbachi et al. reported the outcome of patients with cHL relapsing after allo-SCT who received DLI and/or BV. 66% and 33% of patients received DLI in the BV and non-BV groups, respectively. The probability of being alive and in CR was highest in the group of patients who received BV followed by DLI (40%) compared to 11% in patients who did not receive any, 24% in patients who received DLI only, 21% in patients who received BV only, and 24% in patients who received DLI followed by BV (*p* = 0.003). In multivariate analysis, the use of DLI was found to significantly improved OS [HR 0.51 (0.32-0.83), *p* = 0.007] [68]. There are two main studies assessing the use of CPI after allo-HCT. Herbaux et al. studied the outcomes of 20 patients (19 who were evaluable) relapsing after allo-HCT, who received Nivo as salvage therapy. After Nivo initiation, GvHD occurred in six (30%) patients, all of whom, interestingly, had a prior history of aGvHD. The time between allo-HCT and Nivo initiation was significantly shorter in these patients. The ORR was 95%, with CR and PR rates of 42% and 52%, respectively. Compared with standard options for this indication, PD-1 blockade with Nivo provides durable disease control after allo-HCT with a probability of PFS at 12 months of 58%, and OS of 79%, with an acceptable safety profile [69]. Haverkos et al. also evaluated the use of PD-1 blockade in 31 cHL patients relapsing after allo-HCT. The majority were given Nivo. Seventeen (55%) patients developed GvHD after initiation of anti-PD-1 mAb. The cumulative incidence of grade 3–4 aGvHD was 20% and 17% for severe cGvHD. Of note, GvHD occurred early after initiation of anti-PD-1 mAb after a median of two doses. Unlike the previous study [69], five patients had no history of GvHD prior to anti-PD-1 mAb initiation. There were eight (26%) GvHD-related deaths. Anti-PD-1 therapy resulted in a high ORR (77%), comprising 50% CR and 27% PR. At the last follow-up, 68% of patients were alive [70]. In conclusion, PD-1 blockade can provide durable disease control and prolonged survival in patients relapsing after allo-HCT, however, at the expense of increased risk of GvHD (30–55%), especially in those with a prior history of GvHD. BV was also evaluated as salvage therapy after MRD or MUD allo-HCT in a large EBMT registry-based study, including 184 patients with R/R cHL. The responses to BV after allo-HCT (CR and PR rates of 29% and 45%, respectively) did not appear to be affected by previous exposure to BV before allo-HCT. At last follow-up, 34% of the original BV cohort were alive and in CR versus 18% in the group that did not receive BV (*p* = 0.003). The combination of BV with donor lymphocyte infusion was associated with the highest probability of being alive in CR at the time of last follow-up [68].

Our choice of treatment for relapse after allo-HCT is to first try BV, either alone or in combination with bendamustine, plus donor lymphocyte infusion (DLI), depending on patient and disease history. In the case of failure, we will use anti-PD-1 mAb as it allows good disease control and, unfortunately, there are few other options for survival. One should definitely be cautious when using anti-PD1 mAb after BV and DLI because of the increased risk of acute GVHD. Badar et al. recently assessed the use of oral ibrutinib, a small molecule drug (and another alternative Bruton’s tyrosine kinase (BTK) inhibitor), in seven patients relapsing after allo-HCT, as an alternative for patients with a prior history of GvHD. Four (57%) patients achieved response (CR *n* = 3, PR, *n* = 1) and, overall, ibrutinib was well-tolerated [71]. These results need to be confirmed in larger trials.

## QoL and long-term side effects

Aside from striving to improve response rates, consideration of QoL is equally important. A Norwegian study compared health-related quality of life (HRQoL), assessed by the Short Form 36 (SF-36), in 456 cHL survivors with 2214 norms from the general Norwegian population. They examined the effect of disease characteristics and treatment on differences in HRQoL and demonstrated reduced HRQoL 3–23 years (adjusted for age, gender and educational status) after curative treatment primarily in physical health including physical functioning, role limitations due to physical problems and general health perceptions, compared to the general Norwegian population. Statistically significant differences were found in general health, physical functioning, role limitations (physical), social functioning and in vitality. Time since diagnosis, types of primary treatment, and relapse, were not associated with statistically significant differences in HRQoL [72].

In the H8 randomized trial, HRQoL data from 935 patients after treatment for early stage cHL showed strain and limitations in all subdomains apart from cognitive functioning, and also reduced motivation. Differences in HRQoL improvement with time were linked to age and sex, but not the type of treatment. Fatigue status at the end of treatment seems to predict subsequent HRQoL [73].

Persistent fatigue represents one of the greatest challenges in cHL and efforts should be made to identify the contributing factors and better describe the patterns of recovery within the various HRQoL domains. The effects of treatment on QoL can really jeopardise a patient’s return to normal life, with familial, social, and professional consequences. There are many guidelines to help with the treatment of cHL resulting from numerous randomized controlled trials, but the patient cure does not just depend on prescribing the right treatment. Emphasis on HRQoL following therapy may inform treatment decisions and long-term survivorship goals. To improve HRQoL, clinicians need to learn more about the impact of treatments on HRQoL. Future research should include prospective, longitudinal randomized trials across both treatments and time. They also need to gain further insight into the course of recovery after cure, considering all aspects of life, not only physical but also functional, social and psychological domains. HRQoL will also be improved by the development of new, more effective but less toxic therapies.

## Summary and concluding remarks

**Fig. 1:**
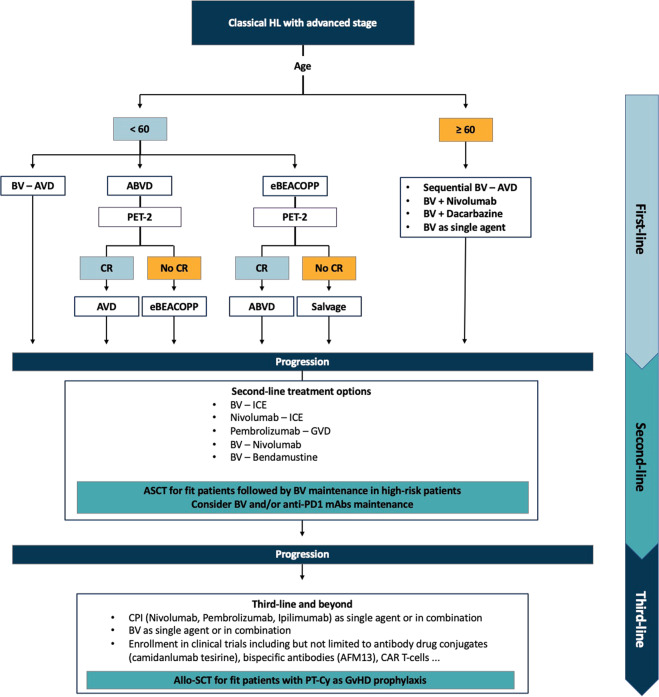
Suggested treatment algorithm for advanced-stage classical Hodgkin Lymphoma. In the frontline setting, patients are treated according to age. In patients aged <60 years, the treatment options can be either immunotherapy (BV) combined with AVD or risk-adapted PET-guided approaches using ABVD and/or eBEACOPP. In patients aged >60 years, the intensity of therapies should be adapted to comorbidities. In the relapsed setting, chemoimmunotherapeutic or chemotherapy-free approaches can be used. ASCT is recommended for fit patients followed by BV maintenance for high-risk patients (a shorter duration of BV or the use of anti-PD1 mAbs alone or in combination with BV may be considered). Beyond third-line therapy, immunotherapy can be used as single agent or in combination. Also, patients can be included in clinical trials using novel agents or cellular therapy. Allo-SCT remains the standard of care for fit and responding patients. PT-Cy should be considered as GvDH prophylaxis for all patients undergoing allo-SCT, even in the non-haploidentical settings. *HL*: Hodgkin Lymphoma; *ABVD*: Adriamycin, Bleomycin, Vincristine, Dacarbazine; *eBEACOPP*: bleomycin, etoposide, doxorubicin (aka adriamycin),cyclophosphamide, vincristine (aka oncovin), procarbazine, prednisolone; *PET*: positron emission tomography; *BV*: brentuximab vedotin; *AVD*: Adriamycin, Vincristine, Dacarbazine; *GVD*: gemcitabine, vinorelbine, liposomal doxorubicin; *CR*: complete response; *ICE*: ifosfamide, mesna, carboplatin, etoposide; *ASCT*: autologous stem cell transplantation; *PD1*: programmed cell death 1; *mAbs*: monoclonal antibodies; *CPI*: checkpoint inhibitors; *Allo-SCT*: allogeneic stem cell transplantation; *PT-Cy*: post-transplant cyclophosphamide; *GvHD*: graft-versus-host disease.

The management of cHL has a huge effect on patients’ QoL not only due to the treatment but also due to the strain and limitations experienced in most aspects of life. In frontline therapy, further individualized intensity with PET-guided therapy may reduce treatment-related morbidity and help prevent relapse by using escalating or de-escalating approaches with ABVD and BEACOPP regimens Another valid frontline option is the combination of BV with AVD, especially in patients who have a contraindication to bleomycin (Fig. 1).

Future perspectives include sequential strategies and the use of anti-PD-1 mAb in combination with AVD. In elderly patients (>60 years), the use of PVAB or the sequential combination of BV-AVD are attractive approaches. Certainly, there is a need for randomized controlled trials to compare the new combination therapies. Regarding second-line treatment, the latest advances show that immunotherapies can safely be combined with chemotherapy followed by consolidation with ASCT in responders. The following treatments showed promising results as first-salvage therapy:

- BV-ICE (ORR 95%, PFS 69%)
- Nivo-ICE (ORR 90%, PFS 79%)
- PEM-GVD (ORR 100%, 1-year PFS 100%)
- BV-Nivo (ORR 85%, PFS 91% after ASCT)
- BV-Bendamustine (ORR 92.5%, PFS 75%)

For patients with a higher risk of relapse, post-ASCT BV maintenance should be given, regardless of remission status or prior BV exposure. Allo-HCT performed after anti-PD-1 mAb is a feasible strategy associated with an excellent PFS and a low incidence of relapse. PT-Cy should be considered for all cHL patients treated with anti-PD1 mAb to reduce the risk of GvHD. Finally, CAR T-cell therapy, not discussed here given the few clinical data to date, but hopefully [52], will also be available and useful for many patients.
